# Why I use both prospective randomized trials and registry data when choosing the personalized treatment of an AAA patient

**DOI:** 10.1007/s00772-018-0434-4

**Published:** 2018-08-09

**Authors:** M. D. Shah, S. Edeiken, R. C. Darling III

**Affiliations:** 1 Division of Vascular Surgery, Albany Medical Center Hospital, Albany, NY USA; 2 The Vascular Group, 391 Myrtle Avenue, Suite 5, 12208 Albany, NY USA

**Keywords:** Observational study, History of medicine, Abdominal aortic aneurysm, Endovascular procedures, Regionalized aortic surgical procedures, Beobachtungsstudie, Medizingeschichte, Bauchaortenaneurysma, Endovaskuläre Verfahren, Regionalisierte Aortenoperationsverfahren

## Abstract

Randomized controlled trials (RCTs) have been the core of level 1 data in medical and surgical science for at least the last three decades. However, frequently patient selection is very narrow, anatomic criteria do not match real-world experience, and much of the work is done in selected academic centers. We use RCTs to help explain the rational for intervention and then rely on longitudinal registries and single center data to give the patients a real-world expectation concerning outcomes and complications in our hands.

## Zusammenfassung

Randomisierte kontrollierte Studien (RCTs) bildeten mindestens drei Jahrzehnte lang die Basis für Daten der Evidenzklasse 1 in den Wissenschaftsbereichen Medizin und Chirurgie. Jedoch ist die Patientenselektion häufig sehr begrenzt, die anatomischen Kriterien entsprechen nicht den realen Erfahrungen und die Hauptarbeit wird in ausgewählten akademischen Zentren geleistet. Auf diese Weise tragen RCTs auch zu den Schwierigkeiten der Daten aus früheren Beobachtungsstudien bei. Wir verwenden RCTs, um die Begründung für einen Eingriff zu erklären, und vertrauen dann auf Längsschnittregister- und Single-Center-Daten, um den Patienten realistische Erwartungen bezüglich den Ergebnissen und Komplikationen in unseren Händen zu geben.

## Introduction

Although surgical science evolved from longitudinal observational experiments, prospective randomized controlled trials (RCTs) have become the level 1 data source for surgical care for at least three decades. The RCTs have pitfalls that are discussed here. Still, many of us use RCT data as a platform to discuss treatment options for patients with abdominal aortic aneurysm (AAA), but we also tend to use observational studies and our own registry data to guide personalized care for each of our patients. The RCTs were the main methodology creating data-driven algorithms. Historically, if observational studies were used to drive patient care then randomization, selection bias, accounting for significant comorbidities, and other metrics were not considered.

Randomized controlled trials were considered to be more reliable

This brought about the concept of randomized controlled trials which were considered to be more reliable and reproducible than observational studies, had data sets that were preconstructed, randomized patients to theoretically remove selection bias, and had power in the studies to prove a hypothesis [1]. The perplexing question in reference to treating individual patients is how to take these data either from a randomized prospective trial or a longitudinal registry, even a large single series experience, and apply them to your patient’s present condition. The RCTs are the gold standard level 1 data on which we like to rely; however, frequently patient selection is very narrow, anatomic criteria do not match real-world experience, and much of the work is done in selected academic centers. This is how RCTs also contribute to the pitfalls of data from historical observational trials.

Alternatively, one can use large series or registry data to guide patient treatment. These allow you to use a broader scope of patient population and get more a real-world experience information; however, many of the variables are uncontrolled and the follow-up, anatomic and demographic data are incomplete. In reality, many of us use a segment of both types of analyses and try to correlate them to our patient’s current situation. To best understand this predicament, one needs to look at the historical aspects of how we have come to the present algorithm of analysis for scientific analysis.

Much of the history of surgical research can be dated back to the nineteenth century when all data were collected and analyzed observationally.

Joseph Lister, the father of surgical antisepsis, recognized that nearly every surgical wound became infected and suppurated; he then chose carbolic acid or phenol rather than dry heat to ensure adequate sterilization for surgery to prevent postoperative infections. He developed his theories by performing experiments, examining wounds, and then applying the results clinically. At first the scientific community rejected this. At a meeting of the American Surgical Association, he encountered strong opposition; however, this type of analytic thought, systematic collection and analysis of data was the birth of surgical science [2, 3].

Joseph Lister recognized that nearly every surgical wound became infected and suppurated

In the mid-nineteenth century, Bernhard von Langenbeck, Professor of Surgery at the University of Berlin, created the first surgical and research training program in Europe. He was the founder of the first journal dedicated entirely to surgical research, Archiv für klinische Chirurgie, and taught other renowned surgical researchers, such as Theodor Billroth, Friedrich Trendelenburg, and Emil Theodor Kocher. Later in North America, the first surgical research training programs were developed at the Johns Hopkin Medical School in 1886 by William Halsted.

One cannot forget John Hunter’s contributions to understanding anatomy and developing new operations; however, it was not until Emil Theodore Kocher from Switzerland became the first Nobel Laureate surgeon rewarded for his study of hypothyroidism-related myxedema that surgeons were recognized for their scientific contributions. He personally performed over 2000 thyroidectomies and a further 7000 were performed at his clinic. In 1909, the Nobel Prize was awarded to him for his work on the physiology, pathology, and surgery of the thyroid. More recently, Alexis Carrel was awarded the Nobel Prize in Physiology and Medicine in 2012 for his numerous contributions, most notably his vascular anastomotic technique and cold temperature preservation of artificial organs. He was able to preserve a feline thyroid gland via cold perfusion for 18 days. Later he achieved preservation of a heart and other organs for several days. Interestingly, he had worked with Charles Lindbergh, the famous aviator, to build a new organ perfusion apparatus capable of supporting heart function while the surgeon assessed the mitral valve. Lindbergh became involved because a relative of his suffered from mitral stenosis and was looking for a better treatment. Frederick Banting was awarded the Nobel Prize in 1923 for his discovery of insulin. A German physician, Werner Theodor Otto Forßmann won the Nobel Prize in 1956, shared with Andre Cournaud and Dickenson Richards, for his work on cardiac catheterization. Forßmann as a surgical resident performed a cardiac catheterization on himself through his basilic vein and was thoroughly chastised by his department chief who called him a clown, which led to an exhaustive investigation, eventually resulted in his winning the Nobel Prize. Charles Huggins won the Nobel Prize in 1966 for the discovery of hormonal therapy for the treatment of prostate cancer and Joseph Murray, a plastic surgeon, was awarded the Nobel Prize in 1990 with E. Donnell Thomas for his discoveries in the field of organ and cell transplantation [3].

Many surgical scientists had to endure criticism, humiliation, and resistance for their innovations. There are likely many others in surgical research over the years that deserved great appreciation but because of political resistance were under recognized. The constant themes for all of these surgical scientists are diligence, persistence, attention to detail, objective data collection, impeccable analysis of data and most importantly making sure that the data was reproducible. This is the basis of our observational research that many of us have used over the years. Unfortunately, as aforementioned, observational research does not factor in numerous variables. It can be affected by multiple biases in patient selection and treatment choices. Thus, the advent of randomized controlled trials took over as the level 1 gold standard for analysis of medical therapies.

The birth of the RCT is typically dated to 1948 for evaluation by the British Medical Research Council of streptomycin for the treatment of tuberculosis, eloquently described by Dr. Podolsky in his article in the New England Journal of Medicine. It was noted that much earlier in 1753 a Scottish surgeon, James Lind published a controlled trial demonstrating that a diet including citrus fruit was effective against scurvy in sailors [4], which has become common knowledge now knowing why sailors were subsequently called limeys.

Prior to the advent of RCTs, there was a system of alternate allocation that studied results after alternating between a treatment and essentially a placebo for a given pathology. Unfortunately, many discovered that selection bias by the surgical scientists could result in equivocal or non-reproducible data.

Selection bias by surgical scientists could result in equivocal or non-reproducible data

For example, some authors admitted to “unconscious selection” when deciding to treat sicker subjects with serum rather than placebo for pneumonia.

In 1962 the US Congress passed the Kefauver-Harris amendments to the Federal Food and Drug and Cosmetic Act to recognize that RCTs had become the expected methodology by which pharmaceutical manufacturers could demonstrate therapeutic safety and efficacy for drug approval. In 1970, the Food and Drug Administration (FDA) required pharmaceutical manufacturers to submit RCT results with new drug applications in order to get approval [4]. The RCTs submitted for drug approval aimed for objectivity and reproducibility. More importantly, they allowed screening of experimental therapies, better data collection, and analysis of confounding variables and contributing biases long before becoming broadly distributed treatments. More recently RCTs have been followed by post-market registries. These were created for many reasons: some for fiscal motives for the companies, some to assess “real-world” data. Since many of the RCTs were very strict in their recruitment criteria, they were not found to be as applicable to the general practice of medicine. Furthermore, many randomized prospective trial expectations and results were too complex, expensive and difficult to apply to everyday clinical scenarios.

High quality registries can answer many of the questions posed by RCTs

Alternatively, high quality registries which have standardized data collection, external audit, and mandatory enrolment can answer many of the questions posed by RCTs at less cost and more than likely, higher enrolment.

Recently, the randomized registry trial concept was described by Michael Lauer and Ralph D’Agostino in the New England Journal of Medicine in 2013. They noted that randomized trials suffered from excessive complexity, expense, time required recruiting study participants, and inadequate representation [5]. The RCTs are possibly designed to be powered to support specific results rather than aimed to guide treatment for world patients. Also, these trials tend to be weighted with large academic institution populations rather than the general community at large. A proposed solution could be the randomized registry trial approach in that all patients are placed in a registry, randomized to different treatment modalities, and then the data are analyzed independently. This was done in the TASTE trial that included 29 Swedish, 1 Icelandic and 1 Danish percutaneous coronary intervention centers [6]. The trial looked at efficacy and safety of thrombus aspiration using this randomized registry trial technique while enlisting thousands of patients, allowing more rapid data analysis, avoiding filling out long case reports and forms, and minimizing the postoperative paperwork for their follow-ups. The conclusion was that the “registry” based randomized trial complements the strengths and addresses the weaknesses of the two prominent types of comparative effectiveness research, RCTs and observational registries. By using randomization then collecting data from a registry of a consecutively enrolled unselected population, the trial was inexpensive, investigators could enrol large numbers of patients resulting in massive data sets, and they could offer clinicians quicker and cheaper insights into a representative sample of real world patients.

Getting back to the intention of this article, how does one use RCTs in conjunction with registries in the practice of vascular surgery to treat AAAs? The RCTs are still the gold standard. It must be recognized that RCTs involve highly selected patients, use narrow or specific treatment indications, take a long time to plan and complete, and are expensive. These issues suggest that RCTs have limited utility for guiding treatment of real-world patients.

RCTs have limited utility for guiding treatment of real-world patients

Registries on the other hand include large unselected consecutive cohorts, are inexpensive to maintain, and contain information on real world patients and outcomes. Registries become deficient when variables or data points are missed, physician or treatment biases without randomization, and uncontrolled confounding factors that affect outcomes. Furthermore, if RCTs and/or registries are supported by industry, there may be some biases, intentional or not, in favor of that industry’s product use rather than contributing to objective investigation.

A comparison was made between the DREAM trial and the EURO Star Registry, an excellent paper on the impact of study design and the outcome of AAA repair. In this comparison, the authors noted that for RCTs with observational non-treatment and treatment arms were underpowered to analyze the data on an intent-to-treat basis and therefore failed to really answer the original questions: when and how do you repair an AAA? During many of the well-known AAA trials, to be discussed below, between 27% and 62% of patients crossed over out of the observational arm into the treatment arm of these trials [7–12]. Most of these crossovers were due to vague symptoms that may or may not be attributable to the aortic pathology. The rationales for crossing over were patient anxiety and concern, and surgeons being uncomfortable waiting any longer to treat AAA patients over extended period after many office visits [10]. The strongest reasons for crossover were patients becoming “symptomatic” of their AAAs and patients requesting AAA repair. Unfortunately, these reasons are subjective and accounted for over three quarters of the patients who left the observational arm in these trials. With that said, it is therefore difficult for a vascular surgeon to objectively evaluate what is deemed the level 1 data from these RCTs.

In the UK Small Aneurysm trial, of the 527 patients randomized to surveillance, 327 subjects crossed over to the open repair by the end of the almost 5‑year mean follow-up period, which means that less than 38% of patients actually remained in their randomized arm. A significant amount of these patients crossed over because of symptoms rather than an increase in aneurysm size [8–12]. Similarly, in the ADAM trial during the 4.8-year mean follow-up period, 62% of those randomized to observation crossed over to open repair. Again, similar reasons were discussed for patients crossing over: concern about vague abdominal pain, patient anxiety, and request for treatment [9]. In the EVAR II trial, about 27% of patients crossed over from observation to undergo AAA repair. Even in the endovascular AAA repair (EVAR) arm, 8% died before receiving EVAR and almost half were from aortic rupture [10–15]. This was beautifully summarized in a paper by Buckley et al. where they concluded, “This inherent weakness has commonly resulted in these trials not resolving to the satisfaction of many if not most clinicians the issue at which they were directed” [13].

How does one use level 1 RCT data in conjunction with longitudinal studies, single center studies and registries? In our group, we use the RCTs to create a general guideline for when to intervene with patients with AAA and a platform for informed discussion with patients. We use the data to support methods for adequate observation with aggressive longitudinal follow-up and to get a rough idea of when EVAR and/or open AAA repair should be considered reasonable, such as for patients with abdominal pain or growing aneurysms or who have barriers to follow-up or treatment.

In our practice, because we provide care for a large rural area in upstate New York, many patients live remote from a major hospital or have difficulty with travel or even cannot take time off from work in order to come for follow-up visits. Despite our having regional offices to provide local office-based care and observation of these patients, many of them want definitive therapy that would protect them from the lethal complication of a ruptured AAA. The RCTs such as the DREAM trial, EVAR I, ADAM, and the UK Small Aneurysm trial have given us enough data to know how often patients’ aneurysms will rupture and at what size, but all of this is based on mandated meticulous follow-up to minimize the patient risk. For the rural populations of our practice, this aggressive observation strategy might not be possible.

These data give us the resources to talk to the patients about what to expect of an AAA and give them the option of relatively early intervention versus long-term observation. We can discuss with them about the PIVOTAL trial as well as the CEASAR trial which did not show any survival benefit from early intervention with EVAR for small aneurysms [16, 17]; however, if one looks at it from the patient perspective, one could also interpret that early intervention did not cause major morbidity or mortality for those who were medically appropriate and for those patients who accepted the risks of AAA surgery, the chance of secondary rupture was relatively low; significant when considering that rural patients may not follow-up aggressively after surgery either. Obviously, choosing who to treat relatively early depends on overall patient condition, the aortic anatomy, suitability, and following the indications for use (IFU) of aortic endografts.

I think in order to give the patients the best perspective of what the options are and what to expect from observation or intervention, we need to use a combination of RCTs and observational trials. One of the most important things that is potentially overlooked when reviewing the level 1 data is that every vascular surgeon should know their own outcomes and their limitations, and should have an objective, honest discussion with their patients and families. They need to know what to expect from the procedure, from minor details to possible major complications. They need to know what the morbidity and mortality is for specific institutions, and even the particular surgeon’s outcomes, in order for patients to make an informed decision. We use longitudinal or observational data, especially from our own institutions and surgeons, to demonstrate to the patients when to treat an AAA, how to treat it whether by EVAR or open repair, and what to expect as far as morbidity and mortality are concerned.

We maintain pre-postoperative follow-ups for these patients in their local environment

While working at over 16 hospitals in our rural catchment area, we have found that for certain hospitals where surgeons performed open AAA repair in the late 1990s and early 2000s, the mortality rate was exceedingly high compared to results from our larger volume institutions for aortic surgery. Thus, we have limited doing open aortic surgery and even EVAR to hospitals that agreed to build the operating room and postoperative care infrastructure needed to make these procedures efficacious for the surgeon and safe for the patient. This type of regionalization allows for best resource utilization and optimizes perioperative outcomes; however, regionalization of AAA repair does mean that the patient and family must travel for the operation and hospital stay, which can be prohibitive, might disrupt social support networks, and temporarily removes patients from their known medical providers. To obviate most of these concerns, we maintain pre-postoperative follow-ups for these patients in their local environments.

By having a travelling office staff and a mobile vascular ultrasound laboratory, our physicians can see patients weekly in rural offices while providing “batched” care regarding doctor’s visits and vascular testing with significantly less disruption of the patient’s circumstances. This may not be possible for every group, but regionalization of healthcare in this fashion has allowed us to maximize the number of patients that can be diagnosed, treated, and followed while maintaining their desired quality of life and limiting uprooting them from their community.

In conclusion, there is no simple answer for how we vascular surgeons use RCTs versus observational studies to guide treatment of patients with AAA; there is no magic bullet. We have discussed the evolution, benefits, and pitfalls of each type of study. There is no simplistic algorithm, therefore one needs to use all of the data objectively first to devise a way to explain to patients and families to understand the disease and treatment options, second to know and explain general outcomes and one’s own outcomes so that expectations can be realistic and met and satisfactory to the patient, and third to maximize patient benefits both socially and medically regarding regionalization and perioperative complications. It is also important to understand the long-term follow-up requirements to adequately observe an AAA and follow-up after repair, especially EVAR, and what it takes to provide mandatory follow-up to patients with geographic, social and medical limitations. We therefore use RTC’s to help explain the rationale for intervention and rely on longitudinal registries and our single center data to give the patients a real-world expectation concerning outcomes and complications.
